# A Lightweight Workflow for Targeted Long-Read Transcriptomic Profiling Using Oxford Nanopore Sequencing

**DOI:** 10.3390/mps9030091

**Published:** 2026-06-04

**Authors:** Mariya Levkova

**Affiliations:** 1 Department of Medical Genetics, Medical University Varna, Marin Drinov Str. 55, 9000 Varna, Bulgaria; mariya.levkova@mu-varna.bg; Tel.: +359-885-692-182; 2 Laboratory of Medical Genetics, St. Marina Hospital, Hristo Smirnenski Blv. 1, 9000 Varna, Bulgaria

**Keywords:** Oxford Nanopore sequencing, long-read transcriptomics, targeted RNA sequencing, FFPE transcriptomics, bioinformatics pipeline

## Abstract

Long-read sequencing technologies provide portable and flexible service, making them attractive for small-scale sequencing studies. However, many existing RNA-sequencing analysis frameworks are designed for transcriptome-wide analyses and require substantial computational resources. Here we present a lightweight and reproducible computational pipeline for targeted long-read transcriptomic profiling using Oxford Nanopore Technologies (ONT) cDNA sequencing data. The pipeline was evaluated using targeted long-read transcriptomic datasets generated from formalin-fixed paraffin-embedded (FFPE) colorectal carcinoma samples previously classified as microsatellite instability—high (MSI-high) by PCR-based testing. Libraries were sequenced on the Oxford Nanopore MinION platform using R10.4.1 flow cells. Application of the workflow enabled rapid quantification of mismatch repair gene expression and detection of immune-related transcripts including CD8A, PDCD1, and HAVCR2 across multiplexed barcode samples. The pipeline performs targeted alignment of long-read sequencing data to a custom transcript reference panel using minimap2, followed by gene-level read counting and normalization using reads-per-million (RPM). Optional modules enable immune marker profiling, detection of reads aligning to multiple genes, exploratory variant analysis, and visualization of expression patterns. By combining simplicity, reproducibility, and minimal computational overhead, the present pipeline provides an accessible framework for targeted transcriptomic analysis of long-read sequencing data. It may facilitate adoption of ONT-based transcriptomic profiling in settings with restricted computational resources.

## 1. Introduction

Long-read sequencing technologies have significantly expanded the scope of transcriptomic analysis by enabling direct characterization of full-length transcripts, alternative splice variants, and complex gene architectures that are difficult to resolve using short-read sequencing approaches [1]. Oxford Nanopore Technologies (ONT) sequencing platforms offer portable and scalable long-read sequencing with relatively low infrastructure requirements, making them attractive for translational and clinical research applications. In particular, the MinION device allows rapid generation of long-read transcriptomic data in laboratory environments without the need for large sequencing facilities [2].

Despite these advantages, bioinformatic analysis of long-read transcriptomic data remains challenging. Many available RNA-sequencing pipelines are designed for transcriptome-wide quantification and typically require substantial computational resources, large reference datasets, and complex statistical modeling frameworks [3]. While such approaches are essential for comprehensive transcriptome studies, they may be unnecessarily complex for targeted gene-panel analyses that focus on a predefined set of genes relevant to a specific biological question [3]. Increasing read length is associated with a larger number of sequenced bases and longer processing times, which can elevate the likelihood of sequencing errors and introduce additional noise into the data [4]. Nevertheless, recent improvements in long-read sequencing technologies have achieved accuracy levels approaching 95% [5], and various error-correction strategies are available to further enhance data reliability [6].

In translational cancer research, targeted transcriptomic profiling is frequently used to investigate pathways of clinical interest [7]. For example, mismatch repair-deficient colorectal cancers, which exhibit microsatellite instability (MSI-high), represent a biologically distinct tumor subtype characterized by high mutational burden and pronounced immune activation. Expression analysis of mismatch repair genes together with immune-related transcripts can therefore provide insight into tumor microenvironment composition and potential response to immunotherapy [8].

Oxford Nanopore sequencing enables full-length transcriptome analysis, allowing comprehensive characterization of transcript structure and gene expression. Previous studies have demonstrated that MinION sequencing can accurately capture full-length cDNA transcripts, with expression levels showing strong concordance with established platforms such as Illumina and PacBio [9]. In translational cancer research, long-read RNA sequencing has been applied across multiple tumor types to investigate clinically relevant transcriptomic alterations, including fusion transcripts, splice variants, and expression signatures associated with therapeutic response. For example, studies in cancer cell lines have demonstrated that long-read sequencing can detect driver fusion events and resolve heterozygous variants using full-length transcript information [10]. Together, these studies highlight the potential of long-read transcriptomic approaches for targeted cancer profiling and support the development of lightweight analytical workflows suitable for exploratory and translational research applications.

Another practical challenge arises from the nature of clinical tumor specimens. Formalin-fixed paraffin-embedded (FFPE) tissue remains the most widely available source of archived clinical samples. However, RNA extracted from FFPE material is often fragmented and degraded, complicating transcriptomic analyses [11]. Lightweight computational workflows capable of processing targeted sequencing data from such samples can therefore be particularly valuable in clinical research settings [3].

To address these needs, we developed msi-longread-panel-light, a lightweight computational workflow for targeted transcriptomic profiling using ONT cDNA sequencing data. The pipeline is designed to quantify expression of predefined transcript panels using simple alignment-based counting and reads-per-million normalization, thereby minimizing computational requirements while maintaining transparency and reproducibility. The workflow supports multiplexed barcode sequencing runs and enables aggregation of expression data across multiple samples.

Here we describe the design and implementation of the pipeline, demonstrate its application to targeted long-read transcriptomic profiling of MSI-high colorectal cancer samples, and discuss its utility for translational cancer research and small-scale sequencing studies.

## 2. Materials and Methods

### 2.1. Sample Collection and RNA Extraction

Formalin-fixed paraffin-embedded (FFPE) colorectal carcinoma samples were obtained from archived clinical material at University Hospital St. Marina, Varna. A total of 10 FFPE colorectal carcinoma samples were included in the study. All samples were reviewed by a certified pathologist prior to RNA extraction. Tumor cell content was assessed on hematoxylin and eosin-stained sections. All analyzed samples demonstrated tumor cell content exceeding 50%, with an estimated average tumor cellularity of approximately 65%. All tumors included in the study had been previously classified as microsatellite instability—high (MSI-high) using the Microsight MSI real-time PCR assay as part of routine clinical diagnostic evaluation. This clinically validated assay provides confirmation of MSI status and supports the biological relevance of the samples analyzed in this study.

Total RNA was extracted from FFPE tissue sections using the RNeasy FFPE Kit (Qiagen, Hilden, Germany; Cat. no. 73504) according to the manufacturer’s instructions. RNA was subsequently purified using silica membrane spin columns and eluted in RNase-free water. RNA concentration was measured using a Qubit fluorometer (Thermo Fisher Scientific, Waltham, MA, USA), and RNA purity was assessed using a NanoDrop spectrophotometer (Thermo Fisher Scientific). Only samples with sufficient RNA concentration (>50 ng/µL) and acceptable purity were included for library preparation. Due to the degraded nature of FFPE-derived RNA, standard integrity metrics such as RIN were not considered reliable and were therefore not used for sample inclusion criteria.

### 2.2. Nanopore cDNA Library Preparation and Sequencing

Complementary DNA (cDNA) libraries were prepared from extracted RNA using the PCR-cDNA Barcoding Kit (SQK-PCB114.24, Oxford Nanopore Technologies, Oxford, UK) according to the manufacturer’s protocol.

Briefly, RNA samples were reverse-transcribed to generate full-length cDNA molecules, followed by PCR amplification and barcode attachment to enable multiplex sequencing of multiple samples within a single sequencing run. No targeted amplification of a predefined gene panel was performed at the library preparation stage. Individual barcoded libraries were pooled prior to sequencing.

Sequencing libraries were loaded onto R10.4.1 flow cells and sequenced using the MinION platform (Oxford Nanopore Technologies, Oxford, UK).

### 2.3. Bioinformatic Workflow

The bioinformatic workflow was implemented in a Conda environment. Basecalling and barcode demultiplexing were performed using Dorado (Oxford Nanopore Technologies, v7.6.8) within the MinKNOW environment (v24.11.10) [12]. Read preprocessing and orientation were performed using Pychopper v2.7.10 [13]. Sequence alignment was conducted using minimap2 v2.30 in long-read mapping mode [14], and alignment processing was performed using samtools v1.22.1 [15]. Additional sequence handling was performed using seqtk v1.5 [16]. Downstream data processing and visualization were implemented in Python v3.12.11 using standard scientific libraries including pandas v2.3.3, numpy v2.3.3, and matplotlib v3.10.6. The resulting basecalled sequencing reads were exported as FASTQ files and organized into barcode-specific directories corresponding to individual tumor samples.

Transcriptomic data were processed using the msi-longread-panel-light pipeline, a lightweight computational workflow developed for targeted transcriptomic analysis of Oxford Nanopore long-read sequencing data.

The pipeline processes basecalled FASTQ files derived from cDNA sequencing libraries and performs gene-level expression quantification using targeted alignment to a curated transcript reference panel.

The workflow includes the following processing steps:

- Merging FASTQ files generated from multiplexed barcode runs;
- Optional orientation of cDNA reads using PyChopper (v2.7.10);
- Alignment of sequencing reads to a custom transcript reference panel;
- Extraction of gene identifiers from alignment output;
- Aggregation of read counts per gene;
- Normalization of gene counts using reads-per-million (RPM);
- Aggregation of results across multiple sequencing runs.

The pipeline was implemented using widely used bioinformatic tools and lightweight scripting components. Core dependencies include minimap2, samtools, seqtk, and Python libraries including pandas, numpy, and matplotlib. Environment management was performed using Conda to ensure reproducibility.

Sequencing reads were merged within barcode-specific directories to generate consolidated FASTQ files representing individual samples. For cDNA libraries, optional preprocessing using PyChopper was applied to orient full-length reads and remove incomplete cDNA constructs.

Standard sequencing quality-control assessment was performed within the MinKNOW/Dorado environment prior to downstream analysis. In addition, basic sequencing summary metrics were monitored at multiple stages of the workflow, including total read counts, read length distribution, and alignment statistics derived from minimap2 output. Given the targeted nature of the reference panel, only a subset of sequencing reads was expected to map to the selected transcripts of interest. Reads with low alignment quality or ambiguous mappings were retained but interpreted cautiously during downstream gene-level aggregation. Multi-mapping reads were not explicitly filtered but were considered in the context of targeted panel design, where gene overlap is limited.

Because FFPE-derived RNA is frequently fragmented and chemically modified, reduced alignment efficiency relative to high-quality RNA datasets was expected. The present workflow was designed as a lightweight proof-of-concept approach for exploratory targeted transcript quantification rather than a systematic evaluation of alignment optimization strategies or comprehensive transcriptome reconstruction.

### 2.4. Targeted Transcript Alignment

The targeted transcript reference panel consisted of a curated set of genes relevant to mismatch repair biology, immune microenvironment characterization, and quality-control markers commonly used in transcriptomic studies. The panel included mismatch repair genes (*MLH1*, *MSH2*, *MSH6*, *PMS2*), immune checkpoint and T-cell markers (*PDCD1*, *CTLA4*, *CD8A*, *CD3D*, *TRAC*), cytotoxic effector genes (*GZMB*, *PRF1*, *IFNG*), macrophage-associated markers (*TYROBP*, *MSR1*, *LST1*, *FCGR3A*), epithelial markers (*EPCAM*), and housekeeping genes (*ACTB*, *GAPDH*, *RPLP0*, *RPS18*).

Reference transcript sequences were obtained from the Ensembl release 115 on the GRCh38 human genome assembly [17]. Transcript sequences corresponding to selected genes of interest were extracted and compiled into a custom FASTA reference panel for targeted alignment. The FASTA headers were formatted to include transcript and gene identifiers to enable gene-level quantification. The use of a restricted reference panel reduces computational requirements and enables rapid quantification of transcripts relevant to the biological context of MSI-high colorectal cancer.

Alignment was performed using minimap2 in long-read mapping mode with default parameters optimized for Oxford Nanopore sequencing data. Given the targeted nature of the analysis and the focus on gene-level quantification rather than base-level variant detection, default parameters were considered appropriate. In addition, low-quality RNA samples were excluded during sample preparation, reducing the impact of degraded FFPE-derived RNA on alignment performance.

Gene identifiers embedded within transcript headers were extracted from the PAF records using lightweight scripting. The number of reads mapping to each gene was counted to generate a raw gene expression table.

Gene counts were subsequently normalized using a reads-per-million (RPM) normalization scheme to allow comparison of expression values across samples with different sequencing depths.

Results from individual barcode samples were aggregated into a combined expression matrix to facilitate downstream visualization and exploratory analysis.

The complete pipeline is distributed as open-source software and is available at

- GitHub: https://github.com/mlevkova/msi-longread-panel-light (accessed on 13 March 2026);
- Zenodo archive: https://doi.org/10.5281/zenodo.18824372 (accessed on 13 March 2026).

### 2.5. Optional Analytical Modules

Several optional analytical modules were implemented to support exploratory analyses of targeted transcriptomic datasets.

An immune profiling module enables extraction of expression values for immune-related genes, including T-cell markers, cytotoxic effector molecules, and immune checkpoint genes. These markers can be used to assess immune activation patterns within tumor samples.

The pipeline also includes functionality for identifying reads that align to multiple genes within the transcript panel. Such reads may represent potential fusion-like events, transcriptional read-through, or complex genomic rearrangements and are reported for further manual inspection.

In addition, the workflow includes an optional module enabling exploratory variant detection using bcftools v1.23. This functionality was included primarily as a proof-of-concept extension of the workflow and was not systematically evaluated in the present study. Given the known limitations associated with FFPE-derived RNA, ONT sequencing error profiles, and RNA-based variant analysis, results generated by this module should be interpreted with substantial caution and are not intended for clinical or high-confidence variant detection.

Visualization scripts implemented in Python enable generation of gene expression heatmaps and expression plots, facilitating rapid interpretation of gene expression patterns across samples.

## 3. Results

### 3.1. Pipeline Application to Targeted Long-Read Transcriptomic Data

The computational workflow was designed to be lightweight and was executed on a standard laboratory workstation. Processing time per sample (barcode) ranged from several minutes to under one hour, depending on sequencing depth and read count.

Two barcode samples (barcode10 and barcode11) exhibited lower alignment counts relative to other samples. This is consistent with reduced sequencing efficiency and potential carryover effects associated with reuse of flow cells.

**Figure 1:**
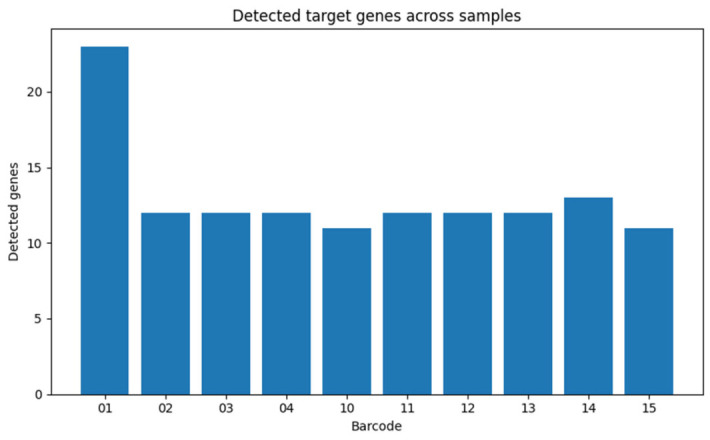
Number of detected target genes across the analyzed FFPE colorectal carcinoma samples. A consistent number of genes (11–13) was detected in the majority of samples, demonstrating robustness of the targeted panel approach despite variability in sequencing depth.

The sequencing datasets analyzed in this study consisted of multiplexed barcode samples generated from FFPE colorectal carcinoma samples previously classified as microsatellite instability—high (MSI-high) using PCR-based diagnostic testing and sequenced on the Oxford Nanopore MinION platform. A total of 10 FFPE colorectal carcinoma samples were included in the final analysis. Sequencing depth varied across samples, with total read counts ranging from approximately 1.3 × 10^5^ to 3.1 × 10^6^ reads per sample. Gene detection was consistent across samples, with the majority showing 11–13 detected transcripts (Figure 1).

Sequencing output varied across barcode samples due to differences in library yield and sequencing performance, which is typical for long-read sequencing of FFPE-derived RNA. Across samples, median read lengths ranged from approximately 250 to 290 bp, consistent with fragmented RNA derived from FFPE material and with cDNA sequencing libraries generated using the ONT PCR-cDNA barcoding protocol.

Multiplexed Oxford Nanopore sequencing runs produced basecalled FASTQ files that were organized into barcode-specific directories corresponding to individual tumor samples. The pipeline successfully processed these datasets through automated read merging, alignment to the targeted transcript reference panel, and generation of normalized gene expression tables.

Across the analyzed datasets, transcripts corresponding to multiple genes included in the targeted panel were detected. These included mismatch repair genes (*MLH1, MSH2, MSH6*, *PMS2*), epithelial markers such as *EPCAM*, and housekeeping genes including *ACTB*, *RPLP0*, *RPS18*, and *GAPDH*. Detection of these transcripts confirms successful capture of RNA molecules from FFPE-derived sequencing libraries and demonstrates the functionality of the targeted alignment and quantification workflow.

The transcript panel used in this study was selected based on biological relevance, including mismatch repair genes and immune-related markers associated with MSI-high colorectal cancer. The panel was intentionally restricted to enable targeted analysis and minimize computational complexity.

**Figure 2:**
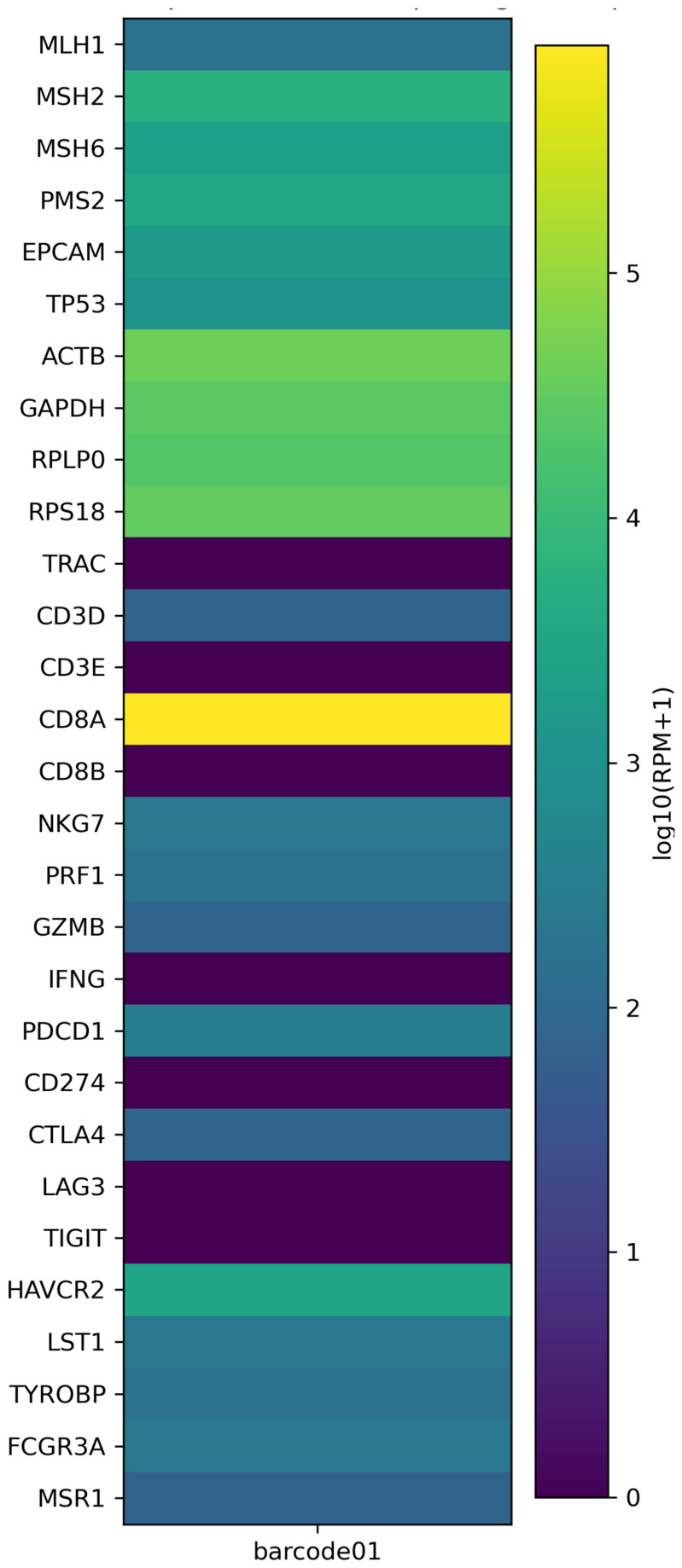
Representative heatmap visualization of targeted gene expression output generated from sample barcode01. Expression values represent log10-transformed reads-per-million (RPM+1) counts derived from Oxford Nanopore cDNA sequencing reads aligned to the custom transcript reference panel. The heatmap illustrates simultaneous detection of mismatch repair genes, housekeeping genes, epithelial markers, and immune-related transcripts including *CD8A*, *PDCD1*, and *HAVCR2*.

**Figure 3:**
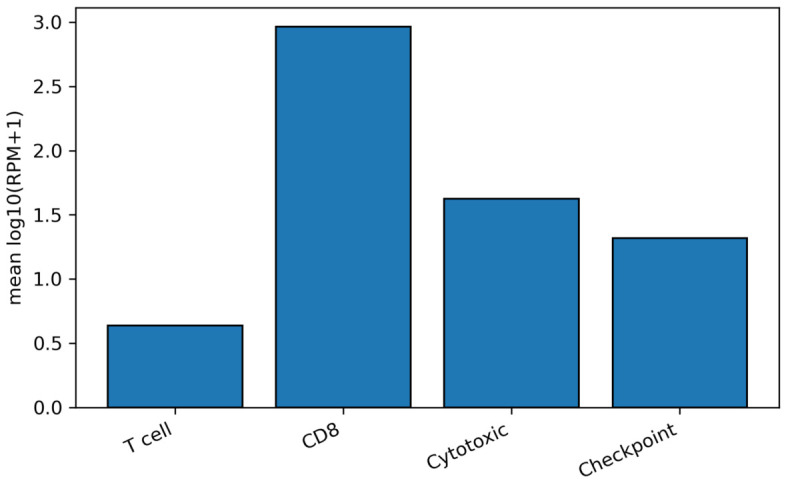
Representative immune-related gene signature output generated from sample barcode01. Signature scores represent the mean normalized expression (log10 RPM+1) of gene groups associated with T-cell markers, cytotoxic effector molecules, and immune checkpoint pathways.

**Figure 4:**
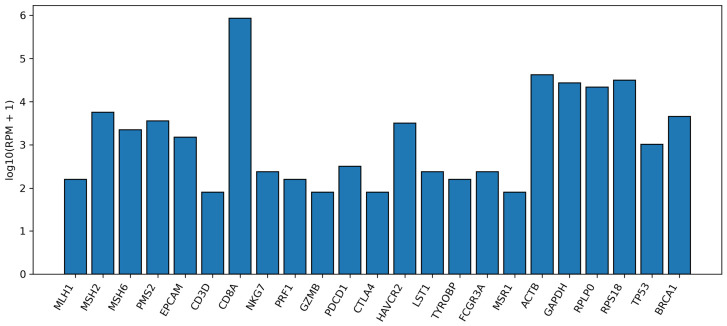
Representative gene-level expression plot generated from sample barcode01. Bars represent log10-transformed reads-per-million (RPM+1) expression values derived from Oxford Nanopore cDNA sequencing reads aligned to the custom transcript reference panel. The panel includes mismatch repair genes, immune-related markers, macrophage-associated genes, and housekeeping transcripts.

For clarity of presentation, Figure 2, Figure 3 and Figure 4 show representative outputs from a single sample (barcode01), while quantitative performance metrics across all samples are summarized in Table 1 and Figure 1. The representative sample (barcode01) was selected based on typical sequencing depth and gene detection metrics consistent with the overall dataset, ensuring that the visualizations reflect standard pipeline output rather than an outlier case.

### 3.2. Visualization of Targeted Gene Expression

To facilitate interpretation of the targeted transcriptomic data, gene expression values were visualized using heatmaps and gene expression plots generated with Python-based visualization scripts.

A representative heatmap illustrating expression of genes included in the targeted transcript panel is shown in Figure 2. The heatmap demonstrates simultaneous detection of mismatch repair genes (*MLH1*, *MSH2*, *MSH6*, *PMS2*), epithelial markers (*EPCAM*), housekeeping genes (*ACTB*, *GAPDH*, *RPLP0*, *RPS18*), and immune-related transcripts including *CD8A*, *PDCD1*, *HAVCR2*, and cytotoxic effector genes.

To summarize immune-related transcriptional activity, expression values for selected immune gene signatures were aggregated into functional categories including T-cell markers, cytotoxic effector genes, and immune checkpoint molecules. The resulting signature scores are illustrated in Figure 3.

A gene-level expression overview for the targeted transcript panel is presented in Figure 4, highlighting expression levels of individual genes detected within the representative dataset.

These visualizations provide a rapid overview of gene expression patterns and illustrate how the pipeline output can be used for exploratory analysis of targeted transcriptomic data.

### 3.3. Pipeline Performance and Output Structure

The msi-longread-panel-light workflow successfully processed multiplexed ONT sequencing datasets using a lightweight computational environment. Alignment and quantification steps were performed using a single-threaded configuration on a standard workstation, demonstrating that targeted long-read transcriptomic analysis can be performed without high-performance computing infrastructure.

For each barcode sample, the workflow generated three primary output files:

- alignment files (*.expanded.paf);
- raw gene count tables (*_counts.tsv);
- normalized expression tables (*_counts_rpm.tsv).

Expression tables from individual samples were subsequently aggregated into a combined dataset (combined_counts_rpm.tsv) to facilitate downstream analysis and cross-sample comparison.

### 3.4. Summary of Pipeline Functionality

Application of the msi-longread-panel-light pipeline demonstrated that targeted long-read transcriptomic analysis can be performed on ONT sequencing datasets derived from FFPE tumor material using a lightweight computational workflow.

The pipeline successfully detected transcripts included in the targeted gene panel, generated normalized expression tables, and produced visualization-ready datasets suitable for downstream exploratory analyses. Variant calling was not performed as part of the primary analysis in this study and is included in the workflow only as an optional exploratory step. Any variant analysis using this approach would require additional validation and higher-quality input data.

This study represents a proof-of-concept implementation of a targeted long-read transcriptomic workflow. While the number of samples analyzed is limited, it is sufficient to demonstrate the functionality and potential applications of the pipeline. Future studies including larger cohorts and publicly available datasets are warranted.

## 4. Discussion

The increasing availability of long-read sequencing technologies provides new opportunities for transcriptomic analysis in translational cancer research. However, many existing computational pipelines are optimized for large-scale transcriptome studies and may introduce unnecessary complexity for targeted gene-panel analyses [3].

Several bioinformatic tools and pipelines have been developed for long-read transcriptomic analysis, including workflows for full-length isoform reconstruction such as Iso-Seq [18] and more complex pipelines integrating alignment, transcript assembly, and isoform detection [19]. In addition, conventional RNA-seq analysis pipelines using splice-aware aligners (e.g., STAR, HISAT2) combined with transcript assembly tools are widely applied for transcriptome-wide studies [20]. These approaches, while powerful, are often computationally intensive and designed for comprehensive transcriptome characterization [21]. In contrast, the present workflow is intentionally lightweight and targeted, focusing on gene-panel-restricted analysis to enable rapid processing and straightforward interpretation, particularly in resource-limited settings.

The msi-longread-panel-light pipeline addresses this gap by providing a lightweight and transparent workflow for targeted long-read transcriptomic profiling. By restricting alignment to a predefined transcript panel and employing simple count-based quantification, the pipeline minimizes computational requirements while maintaining reproducibility and interpretability.

The workflow is particularly suitable for exploratory studies involving limited sample numbers or targeted gene panels. In addition, the ability to process multiplexed barcode runs and aggregate results across sequencing runs makes the pipeline practical for iterative experimental designs commonly encountered in translational research.

The validation experiments presented here demonstrate that the pipeline can successfully quantify gene expression patterns from FFPE tumor samples sequenced using ONT long-read technologies. Detection of immune-related transcripts within MSI-high colorectal cancer samples highlights the potential utility of targeted long-read transcriptomics for characterizing tumor microenvironment signatures [8].

Several limitations of the present study should be acknowledged.

First, the validation dataset used to demonstrate the functionality of the workflow consisted of a relatively small number of tumor samples. Although the purpose of this study was primarily to present a computational pipeline rather than perform a comprehensive biological investigation, larger datasets will be required in future studies to evaluate the performance of the workflow across broader cohorts and diverse tumor types.

Second, sequencing was performed using the Oxford Nanopore MinION platform, which provides portable long-read sequencing but offers lower throughput compared with larger ONT instruments such as the GridION or PromethION. As a result, sequencing depth varied across barcode samples. Although variability related to sequencing depth may influence transcript detection, this aspect was not systematically evaluated in the present study and should be considered in future investigations.

Third, the biological interpretation of gene expression patterns in the present study should be considered exploratory. The datasets were primarily used to illustrate the functionality of the pipeline rather than to draw definitive conclusions about tumor biology or immune microenvironment characteristics. Future studies involving larger cohorts and deeper sequencing will be necessary to fully explore the biological insights that may be obtained using targeted long-read transcriptomic profiling.

In addition, the expression quantification approach implemented in the workflow relies on simple read-count aggregation followed by reads-per-million normalization. While this strategy provides a transparent and computationally lightweight method for estimating transcript abundance, it does not account for transcript length differences, amplification biases introduced during PCR-based library preparation, or potential ambiguity arising from reads that align to closely related transcripts. Consequently, the expression values generated by the pipeline should be interpreted as approximate indicators of relative transcript abundance rather than precise quantitative measurements.

Despite these limitations, the lightweight design of the pipeline makes it accessible to laboratories without extensive computational infrastructure. By providing a reproducible and easily deployable workflow for targeted transcriptomic profiling, the pipeline may facilitate broader adoption of long-read sequencing technologies in translational cancer research.

## 5. Conclusions

In this study, we present msi-longread-panel-light, a lightweight computational workflow for targeted transcriptomic analysis of Oxford Nanopore long-read sequencing data. The pipeline enables rapid gene-level quantification across multiplexed barcode sequencing runs, as demonstrated by consistent detection of target genes across all analyzed samples.

The computational design of the pipeline prioritizes simplicity, transparency, and low resource requirements, allowing targeted transcriptomic analyses to be performed on standard laboratory workstations without high-performance computing infrastructure. The workflow is therefore well suited for small- to medium-scale translational research studies, exploratory sequencing projects, and targeted gene-panel analyses.

## Figures and Tables

**Table 1:** Summary of sequencing and analysis metrics across FFPE samples.

Sample Name	Reads	Aligned Reads	Detected Genes
barcode01	710,777	2041	23
barcode02	1,580,878	3587	12
barcode03	994,012	1917	12
barcode04	3,150,494	7248	12
barcode10	204,398	619	11
barcode11	128,467	536	12
barcode12	1,020,099	3006	12
barcode13	1,443,924	2573	12
barcode14	626,534	1299	13
barcode15	685,678	1643	11

## Data Availability

The bioinformatic workflow described in this study is available as open-source software at https://github.com/mlevkova/msi-longread-panel-light (accessed on 13 March 2026). A versioned archive of the pipeline is available at Zenodo: https://doi.org/10.5281/zenodo.18824372 (accessed on 13 March 2026). The sequencing data analyzed in this study were generated from clinical FFPE samples and are not publicly available due to ethical and patient privacy considerations.

## Abbreviations

The following abbreviations are used in this manuscript:

ACTB	Beta-Actin
AI	Artificial Intelligence
BC	Barcode
cDNA	Complementary DNA
CD3D	CD3 Delta Chain
CD8A	CD8 Alpha Chain
CTLA4	Cytotoxic T-Lymphocyte Associated Protein 4
EPCAM	Epithelial Cell Adhesion Molecule
FFPE	Formalin-Fixed Paraffin-Embedded
GZMB	Granzyme B
HAVCR2	Hepatitis A Virus Cellular Receptor 2 (TIM-3)
IFNG	Interferon Gamma
LST1	Leukocyte Specific Transcript 1
MLH1	MutL Homolog 1
MMR	Mismatch Repair
MSH2	MutS Homolog 2
MSH6	MutS Homolog 6
MSI	Microsatellite Instability
MSI-H	Microsatellite Instability—High
MSR1	Macrophage Scavenger Receptor 1
ONT	Oxford Nanopore Technologies
PAF	Pairwise Alignment Format
PCR	Polymerase Chain Reaction
PDCD1	Programmed Cell Death Protein 1
PMS2	PMS1 Homolog 2
PRF1	Perforin 1
RNA	Ribonucleic Acid
RPM	Reads Per Million
TCR	T-cell Receptor
TRAC	T-cell Receptor Alpha Constant
TYROBP	TYRO Protein Tyrosine Kinase Binding Protein

## References

[1] Monzó C., Liu T., Conesa A. (2025). Transcriptomics in the era of long-read sequencing. Nat. Rev. Genet..

[2] Mock A., Braun M., Scholl C., Fröhling S., Erkut C. (2023). Transcriptome profiling for precision cancer medicine using shallow nanopore cDNA sequencing. Sci. Rep..

[3] Zhang T., Jiang M., Li H., Gao Y., Yousuf S., Yu K., Yi X., Wang J., Yang L., Liu Y.-X. (2025). Computational tools and resources for long-read metagenomic sequencing using nanopore and PacBio. Genom. Proteom. Bioinform..

[4] Ebler J., Haukness M., Pesout T., Marschall T., Paten B. (2019). Haplotype-aware diplotyping from noisy long reads. Genome Biol..

[5] Amarasinghe S.L., Su S., Dong X., Zappia L., Ritchie M.E., Gouil Q. (2020). Opportunities and challenges in long-read sequencing data analysis. Genome Biol..

[6] Baid G., Cook D.E., Shafin K., Yun T., Llinares-López F., Berthet Q., Belyaeva A., Töpfer A., Wenger A.M., Rowell W.J. (2023). DeepConsensus improves the accuracy of sequences with a gap-aware sequence transformer. Nat. Biotechnol..

[7] Chida K., Kawazoe A., Suzuki T., Kawazu M., Ueno T., Takenouchi K., Nakamura Y., Kuboki Y., Kotani D., Kojima T. (2022). Transcriptomic profiling of MSI-H/dMMR gastrointestinal tumors to identify determinants of responsiveness to anti–PD-1 therapy. Clin. Cancer Res..

[8] Gallois C., Landi M., Taieb J., Sroussi M., Saberzadeh-Ardestani B., Cazelles A., Lonardi S., Bergamo F., Intini R., Maddalena G. (2023). Transcriptomic signatures of MSI-high metastatic colorectal cancer predict efficacy of immune checkpoint inhibitors. Clin. Cancer Res..

[9] Oikonomopoulos S., Wang Y.C., Djambazian H., Badescu D., Ragoussis J. (2016). Benchmarking of the Oxford Nanopore MinION sequencing for quantitative and qualitative assessment of cDNA populations. Sci. Rep..

[10] Seki M., Katsumata E., Suzuki A., Sereewattanawoot S., Sakamoto Y., Mizushima-Sugano J., Sugano S., Kohno T., Frith M.C., Tsuchihara K. (2019). Evaluation and application of RNA-Seq by MinION. DNA Res..

[11] Lin Y., Dong Z.-H., Ye T.-Y., Yang J.-M., Xie M., Luo J.-C., Gao J., Guo A.-Y. (2024). Optimization of FFPE preparation and identification of gene attributes associated with RNA degradation. NAR Genom. Bioinform..

[12] Oxford Nanopore Technologies (2026). Dorado Basecalling Software.

[13] Oxford Nanopore Technologies (2026). Pychopper.

[14] Li H. (2018). Minimap2: Pairwise alignment for nucleotide sequences. Bioinformatics.

[15] Li H., Handsaker B., Wysoker A., Fennell T., Ruan J., Homer N., Marth G., Abecasis G., Durbin R., 1000 Genome Project Data Processing Subgroup (2009). The sequence alignment/map format and SAMtools. Bioinformatics.

[16] Li H. (2026). Seqtk; Version v1.5.

[17] Harrison P.W., Amode M.R., Austine-Orimoloye O., Azov A.G., Barba M., Barnes I., Becker A., Bennett R., Berry A., Bhai J. (2024). Ensembl 2024. Nucleic Acids Res..

[18] Sharon D., Tilgner H., Grubert F., Snyder M. (2013). A single-molecule long-read survey of the human transcriptome. Nat. Biotechnol..

[19] Tang A.D., Soulette C.M., van Baren M.J., Hart K., Hrabeta-Robinson E., Wu C.J., Brooks A.N. (2020). Full-length transcript characterization of SF3B1 mutation in chronic lymphocytic leukemia reveals downregulation of retained introns. Nat. Commun..

[20] Pertea M., Kim D., Pertea G.M., Leek J.T., Salzberg S.L. (2016). Transcript-level expression analysis of RNA-seq experiments with HISAT, StringTie and Ballgown. Nat. Protoc..

[21] Marx V. (2023). Method of the year: Long-read sequencing. Nat. Methods.

